# Omalizumab Therapy Results in Defined Behavioural Changes and Improvements in Quality of Life in Solar Urticaria

**DOI:** 10.1111/phpp.70088

**Published:** 2026-04-10

**Authors:** Donna Parkin, Elizabeth J. Marjanovic, Mark D. Farrar, Lesley E. Rhodes, Kirsty J. Rutter

**Affiliations:** ^1^ Photobiology Unit, Dermatology Centre, Salford Royal Hospital, Northern Care Alliance NHS Foundation Trust, Manchester Academic Health Science Centre Greater Manchester UK; ^2^ The Centre for Dermatology Research, Division of Musculoskeletal and Dermatological Sciences, School of Biological Sciences, Faculty of Biology, Medicine and Health, NIHR Manchester Biomedical Research Centre The University of Manchester Manchester UK

**Keywords:** omalizumab, photosensitivity disorders, quality of life, solar, sun exposure behaviour, urticaria

## Abstract

**Background:**

Solar urticaria is a rare photodermatosis in which exquisite photosensitivity can require extreme behavioural adaptations, resulting in a substantial impact on quality of life (QoL). Omalizumab therapy has been demonstrated to improve several clinical outcome measures, but the impact on behavioural measures is poorly understood. Our objectives were to examine daylight exposure behaviours and QoL measures pre‐ and post‐omalizumab therapy.

**Methods:**

Daylight exposure diaries were completed by *n* = 5 patients with solar urticaria and *n* = 7 healthy participants during different seasons in England, UK (51.1–53.5^o^N). These incorporated a range of measures, including time spent outdoors and clothing worn, and Dermatology Life Quality Index (DLQI) data were also collected.

**Results:**

Prior to omalizumab, patients spent less time outdoors in sunny vs. non‐sunny conditions in spring (mean 10 vs. 29 min/day, *p* < 0.05) and less than healthy volunteers (44 min in sunny conditions/day). On omalizumab, patients increased their time outdoors in sunny conditions, reaching similar levels to healthy volunteers in spring (45 min/day) and further increasing to 85 min/day in summer. This was accompanied by fewer days with symptoms (symptoms on 75% days in spring pre‐omalizumab vs. 26% days in summer on omalizumab), an apparent doubling of skin surface area exposure and substantial improvement in patients' QoL (mean past‐year DLQI pre‐omalizumab vs. past‐week summer on omalizumab 22 vs. 5, *p* < 0.001).

**Conclusion:**

Omalizumab therapy was associated with behavioural changes that increase daylight exposure, accompanied by improved QoL. This study highlights the importance of considering a range of outcome measures in assessing response to therapy.

## 1 Introduction

Solar urticaria is a rare photodermatosis comprising a chronic, inducible urticaria provoked by exposure to solar and sometimes artificial sources of radiation [1, 2]. The pathomechanisms are not fully understood but are presumed to involve the development of specific IgE to a photoallergen generated on ultraviolet radiation (UVR) and/or visible light (VIS) exposure. Recent data support the central role of mast cells and IgE in this process, and the release of mast cell cellular mediators leading to the clinical features of wheal and flare [3]. Solar urticaria is associated with a substantial impact on patients' quality of life (QoL), due not only to the often severe symptoms but also to the behavioural adaptations that are frequently required to prevent the provocation of symptoms, such as limiting time spent outdoors or extensively covering the skin [4, 5]. This may result in profound psychosocial and lifestyle implications.

Omalizumab, an anti‐IgE monoclonal antibody, has proven efficacy in the management of chronic urticaria and is licensed for the treatment of chronic spontaneous urticaria [6, 7]. Although it remains unlicensed for chronic inducible urticaria, numerous publications and several systematic reviews support its use in solar urticaria [8, 9, 10]. Several studies have reported improvement in objective parameters such as provocation testing and minimal urticaria doses following omalizumab therapy, while relatively few have examined effects on QoL [11, 12, 13]. Behavioural alterations are also important to assess since patients may have ingrained UVR/VIS avoidance strategies that can be challenging to adjust, but to our knowledge, these aspects have not previously been formally evaluated. We report here the effect of omalizumab therapy on a range of behavioural parameters relating to daylight exposure, and also QoL, in a cohort of patients with solar urticaria receiving omalizumab therapy, and compare with the behaviour of healthy volunteers.

## 2 Methods

An observational study was conducted in Manchester, UK, between June 2014 and March 2023 on a cohort of patients diagnosed with solar urticaria living at latitude 51.1–53.5^o^N. Data were collected prior to and during treatment with omalizumab. Treatment was commenced during spring (March, April, May) and was administered subcutaneously at a dose of 300 mg every 4 weeks for a 6‐month period in all but one patient who commenced omalizumab in summer.

Patients completed daylight exposure diaries for at least one month in each of three seasons: spring (March–May), summer (June–August), autumn (September–November), recording time spent outdoors, weather type, clothing worn, symptom occurrence and sunscreen use. Quality of life was assessed using the Dermatology Life Quality Index (DLQI); higher scores denote greater impact on QoL, with scores > 10 reflecting very or extremely high impact [14]. Where data were collected over several years, average (mean) values were calculated. Given the difficulty inherent in capturing QoL impact of an intermittent, seasonal and behaviour‐dependent condition, past‐week and past‐year DLQI scores were obtained; while presently unvalidated, past‐year DLQI scores can be used to overcome these difficulties and have been described by several authors [4, 5]. Healthy control participants also completed the daylight exposure diaries for a period of one month during each season.

The daylight exposure diary was a modification of that successfully used in published studies of daylight exposure (including in a group of patients with a range of photosensitivity disorders) [15, 16]. The diary comprises a time‐scale from 6 am to 8 pm divided into 15 min blocks where participants indicate when they are outdoors and the prevailing weather conditions: Variable, Overcast, Rain or Sun. For the purposes of this study, the data for Variable, Rain and Overcast weather types were combined to give a binary outcome: No Sun or Sun conditions. The diaries also allowed patients to record clothing type (long or short sleeves/trousers/skirts) when outdoors and if a hat or head‐covering was worn. The percentage skin surface area (SSA) exposed when outdoors was estimated from information on clothing worn as previously described [16].

Patients were diagnosed with solar urticaria following full photobiological investigation, which included detailed history and examination, DLQI scoring, monochromator phototesting, broadband UVR provocation testing, photopatch testing, blood and urine porphyrin scans, connective tissue disease screen, immunoglobulin E (IgE) levels and vitamin D status. To determine the action spectrum and minimum urticaria doses (MUDs) using monochromator phototesting, skin on the mid‐back was exposed to narrow bandwidths of UVB, UVA and VIS using a 300 W xenon light source with a built‐in filter wheel (Asahi Spectra USA). Incremental dose series were used for exposures to 300, 320, 330, 350, 370, 400, 500 and 600 nm (half‐maximum bandwidth of 10 nm at 300–350 nm, 15 nm at 370–400 nm and 25 nm at 500–600 nm). Photoprovocation testing was performed on 5 × 5 cm areas of ventral forearm skin. One forearm was exposed to 15 J/cm^2^ broadband UVA (320–400 nm) using a custom‐built unit incorporating Cleo Performance bulbs (Philips Healthcare UK Ltd). The contralateral forearm was exposed to 10 J/cm^2^ solar‐simulated UVR (290‐400 nm) with a 1‐kW xenon arc lamp plus an atmospheric attenuation filter (Newport Spectra‐Physics Ltd). These doses represent physiological exposure approximating 30 min of local midday summer sun on a clear day in the UK.

The behavioural study was approved by the Greater Manchester West Research Ethics Committee (Ref 18/NW/0494), and patients gave written consent for the use of their routinely collected clinical data.

## 3 Analysis

Data were analysed using descriptive statistics using GraphPad Prism version 9 (GraphPad Software, Boston, USA). Comparisons used paired (within group) or unpaired (between groups) *t*‐tests or non‐parametric equivalents. Two‐tailed *p* < 0.05 was considered statistically significant. Mean values for time spent outdoors, percentage SSA exposure and DLQI were calculated prior to and following administration of omalizumab for each treatment year. The yearly values were then averaged for each patient.

## 4 Results

### 4.1 Patient Characteristics

Five patients with severe solar urticaria (3 M, 2 F, mean age 39 years; skin phototype I‐III) completed diaries before and during omalizumab treatment (Table 1). Assessment of severity was based on the very high DLQI scores (range 19–27) and severely abnormal phototests, with very low MUDs. Patients showed a range of action spectra; all showed exquisite photosensitivity and rapid provocation by broadband and narrowband UVR sources. All patients had been inadequately controlled by updosed antihistamines, and three had tried phototherapy with limited/no benefit. One patient continued antihistamines whilst taking omalizumab. Seven healthy volunteers also completed the same behaviour/exposure diaries (2 M, 5 F; mean age 47 years; skin phototype I‐III).

**TABLE 1:** Clinical characteristics of patients.

Patient characteristic	Patient 1	Patient 2	Patient 3	Patient 4	Patient 5
Age at diagnosis	30	48	20	30	53
Sex	M	F	M	M	F
Duration of SU	8 months	7 years	2 years	15 months	4 years
Skin phototype	I	II	III	III	II
Ethnicity	White British	White British	White British	White British	White British
Action spectrum on monochromator testing	320–500 nm	320–350 nm	320–600 nm	400–500 nm	300–400 nm
Solar simulated UVR provocation	Immediate wheal and flare	Immediate wheal and flare	Immediate wheal and flare	Immediate wheal and flare	Immediate wheal and flare
Broadband UVA provocation	Immediate wheal and flare	Immediate wheal and flare	Immediate wheal and flare	Negative	Immediate wheal and flare
Baseline IgE, kU/L (normal range < 114)	657	13	432	642	322
25‐hydroxyvitamin D, nmol/L (normal range 50–125)	24.4	42.5	36.6	14.8	21.4
Baseline DLQI (past‐year)	19	27	26	20	20
Data collection period	6 years	4 years	2 years	2 years	2 years

**FIGURE 1:**
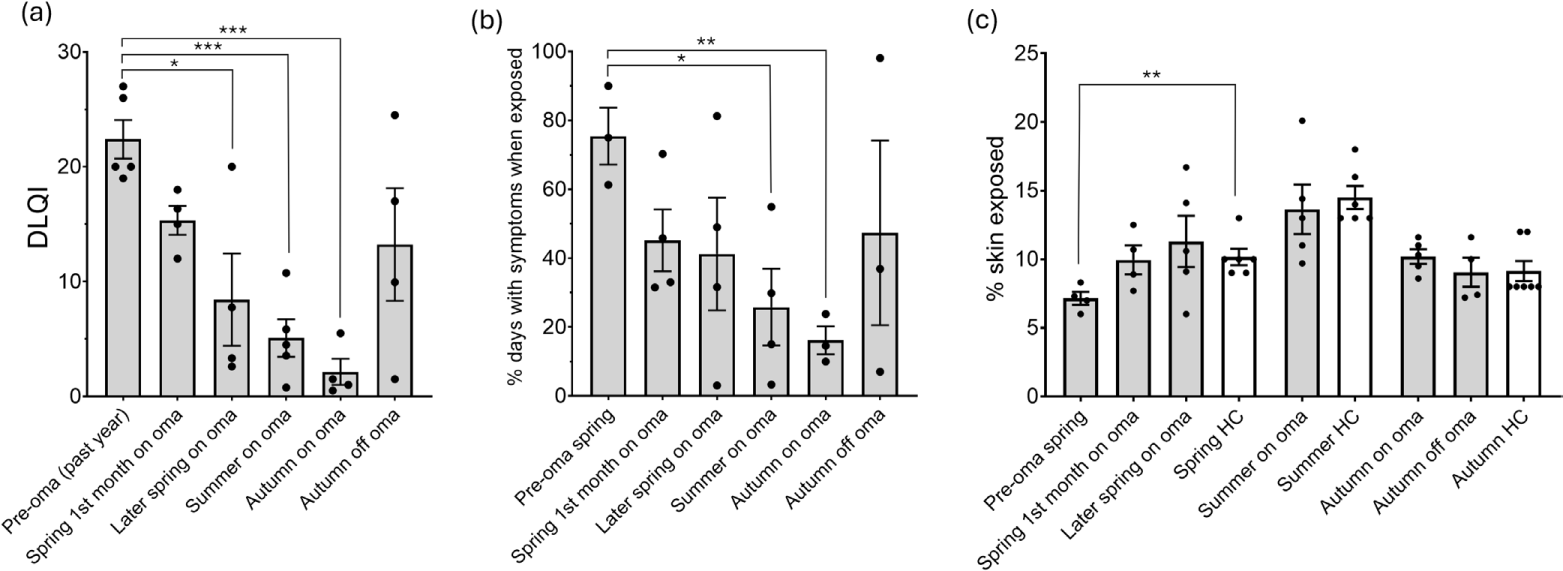
(a) Mean Dermatology Life Quality Index scores before and during omalizumab therapy in patients with solar urticaria. (b) Mean percentage of days with symptoms when outdoors before and during omalizumab therapy. (c) Mean percentage skin surface area exposed in patients with solar urticaria before and after omalizumab therapy (grey bars) and in healthy volunteers (white bars). Data are mean and SEM; *p < 0.05, **p < 0.01, ***p < 0.001. Oma = omalizumab; HC = healthy volunteers.

Following omalizumab therapy, there was a substantial and statistically significant improvement in patients' QoL as measured by DLQI (Figure 1a; past‐year without omalizumab vs. summer on omalizumab mean 22.4 vs. 5.1, *p* < 0.001). This was accompanied by a reduction in the occurrence of symptoms, with symptoms occurring 75.4% days in spring when outdoors pre‐omalizumab vs. 25.8% days in summer on omalizumab (*p* < 0.05; Figure 1b). Patients exposed significantly less skin surface area than healthy volunteers in spring prior to omalizumab therapy (7.2 vs. 10.2%, *p* < 0.01) (Figure 1c). However, after commencing omalizumab, the skin surface area exposed to sunlight appeared to increase, with an approximate doubling of the area exposed in summer on omalizumab therapy vs. in spring without omalizumab (13.6 vs. 7.2%, *p* = 0.07), and reaching a similar percentage exposure to healthy volunteers in spring, summer and autumn. Three patients also found that they were able to utilise self‐hardening with natural sunlight in spring/summer whilst on omalizumab, having previously been unable to manage this due to the severity of their photosensitivity, and this appeared to augment the benefit of therapy. Sunscreen use was variable and did not show a significant change with omalizumab therapy. After discontinuing omalizumab in autumn, DLQI scores worsened and the percentage of days with symptoms increased again (Figure 1a,b).

**FIGURE 2:**
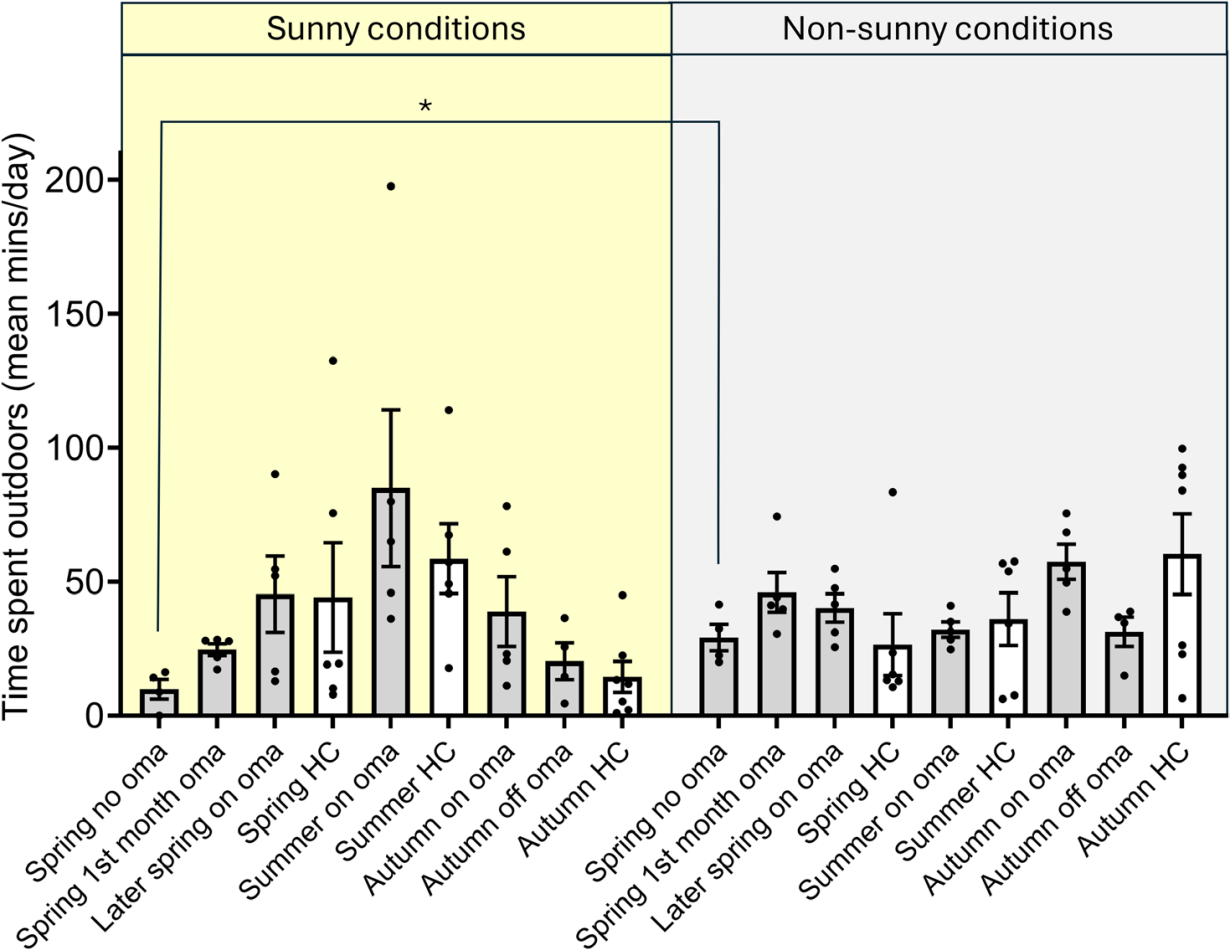
Time spent outdoors in sunny conditions in patients with solar urticaria (grey bars) before and after omalizumab therapy, and in healthy volunteers (white bars), in sunny (yellow panel) and non‐sunny (grey panel) conditions; data are mean and SEM, *p < 0.05. Oma = omalizumab; HC = healthy volunteers.

Prior to omalizumab, patients spent significantly less time outdoors in sunny vs. non‐sunny conditions in spring (10 vs. 29 min/day, *p* < 0.05, Figure 2). This contrasted with healthy volunteers, who appeared to spend more time in sunny vs. non‐sunny conditions (44 vs. 26 min/day, difference non‐significant). During omalizumab therapy, patients were progressively able to increase the amount of time spent outdoors in sunny conditions, spending a similar amount of time in sunny conditions in spring as healthy volunteers (45 vs. 44 min/day) and increasing to 85 and 39 min/day in summer and autumn, respectively. After discontinuing omalizumab in the autumn, the time spent outdoors in both sunny and non‐sunny conditions decreased.

**FIGURE 3:**
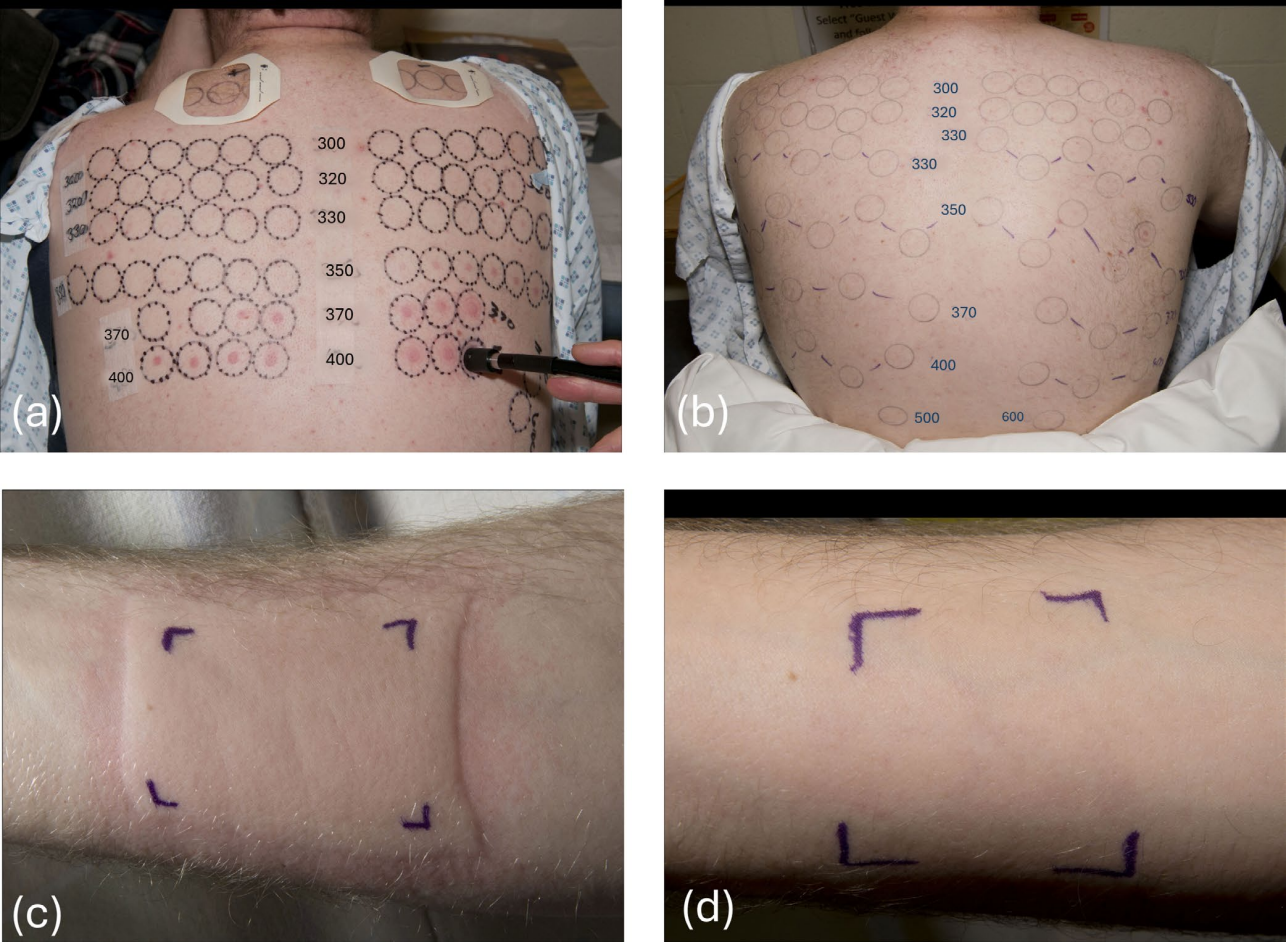
Representative images of monochromator testing (a) pre‐omalizumab, showing urticarial reactions between 330 and 400 nm, (b) during omalizumab therapy, showing no urticarial reactions, and broadband UVA provocation testing (c) pre‐omalizumab, showing a clear wheal‐and‐flare response, (d) during omalizumab therapy, showing no reaction.

Monochromator testing prior to omalizumab therapy showed urticaria readily provoked by low doses of UVR and often also VIS. Following omalizumab therapy, repeat monochromator testing 4–8 months later showed substantial improvements, with three patients negative for all wavelengths tested (Figure 3a,b, Table 2). Of the two patients still showing urticarial provocation, the MUDs had improved, although in one patient, the MUDs improved at 4 of the 6 urticaria‐provoking wavelengths and were unchanged at 2 wavelengths (350, 370 nm). Fold change in individual MUDs ranged from 2 to > 125. Broadband provocation testing to broadband UVA and solar simulated UVR was also repeated in two patients, and urticaria could no longer be provoked (Figure 3c,d).

**TABLE 2:** Minimal urticarial doses prior to and during omalizumab therapy.

Wavelength (peak)	Highest dose tested (J/cm2 except mJ/cm2 at 300 nm)	MUD (J/cm2 except mJ/cm2 at 300 nm)														
		Patient 1			Patient 2			Patient 3			Patient 4			Patient 5		
		Pre‐oma	On oma	Fold change MUD	Pre‐oma	On oma	Fold change MUD	Pre‐oma	On oma	Fold change MUD	Pre‐oma	On oma	Fold change MUD	Pre‐oma	On oma	Fold change MUD
300	80	Neg	Neg	—	Neg	Neg	—	Neg	Neg	—	Neg	Neg	—	3.5	40	11.4
320	4	2.5	Neg	> 1.6	0.7	Neg	> 5.7	1	Neg	> 4	Neg	Neg	—	0.007	0.63	90
330	14	5	Neg	> 2.8	0.9	Neg	> 15.6	0.63	Neg	> 22.2	Neg	Neg	—	0.025	0.05	2
350	40	3.5	Neg	> 11.4	1.3	40	30.8	0.31	Neg	> 129	Neg	Neg	—	0.63	0.63	—
370	57	3.5	Neg	> 16.3	1.8	Neg	> 31.7	Neg	Neg	—	Neg	Neg	—	0.22	0.22	—
400	113	0.9	Neg	> 125.6	0.9	113	125.6	Neg	Neg	—	14	Neg	> 8.1	0.9	7	7.8
500	50	6.3	Neg	> 7.9	6.3	Neg	> 7.94	Neg	Neg	—	13	Neg	> 3.8	neg	neg	—
600	50	Neg	Neg	—	50	Neg	> 1	Neg	Neg	—	Neg	Neg	—	neg	neg	—

Abbreviations: MUD, minimal urticarial dose; oma, omalizumab.

## 5 Discussion

The anti‐IgE agent omalizumab is a recognised therapy for solar urticaria and is included within international guidelines as a second‐line therapy for chronic urticaria, if there is inadequate response to antihistamines [7], although it remains currently unlicensed specifically for inducible urticarias. Measuring outcomes of therapy in inducible urticarias can be challenging, but one can employ both subjective and objective measures. While previous studies have reported benefits from omalizumab use in solar urticaria, measures of “response” have varied [8, 10]. These have included QoL measures and improved phototest findings, though several have been limited to reporting less detailed outcomes, such as “remission” and “complete response.” This study by contrast has analysed detailed behavioural factors particularly relevant to patients with solar urticaria, including time spent outdoors in a range of weather conditions, symptom occurrence and percentage skin surface area exposed based on clothing worn, which to our knowledge have not been previously evaluated. We now demonstrate that omalizumab therapy led to substantial increases in time spent outdoors by patients, which reached similar levels to healthy volunteers, fewer days with symptoms and increased skin surface area exposure, accompanied by improvements in objective QoL measures. These findings show that omalizumab therapy is associated with changes in what patients actually do in daily life, as well as symptom and QoL improvement, and these changes could have substantial impacts on social and emotional wellbeing and potentially on physical activity.

Behavioural change following omalizumab therapy is especially important to consider given the exquisite, severe, broad‐spectrum photosensitivity experienced by many patients. This can result in extreme restrictions on daily life, since symptoms are often provoked by only a few minutes of daylight exposure, which often occurs year‐round, even in a seasonal climate, owing to photosensitivity to ambient levels of longer wavelength UVA and VIS [17]. It can be challenging for patients to develop the confidence to spend more time outdoors and adjust their clothing choices, having previously experienced severe discomfort from urticarial provocation. Notably, our data demonstrate that patients can succeed in progressively increasing their time outdoors in sunny conditions following omalizumab therapy, reaching or even exceeding the time spent by healthy people. These improvements were mirrored by increases in percentage skin surface area exposed to daylight and fewer days with symptoms when outdoors. These behavioural alterations could lead to significant improvement in patients' physical, social and emotional wellbeing through enhancing the ability to spend time outdoors and participate in “normal” outdoor activities without experiencing distressing symptoms. Increased time spent outdoors and daylight exposure have been associated with improved mood and physical activity in the general population in adults and young people, respectively [18, 19]. A further anticipated benefit could be increased potential for vitamin D synthesis through the ability to be exposed to low‐dose, vitamin D‐generating levels of UVR [20]. We did not find any change in sunscreen use following the use of omalizumab therapy, but in general, even if they do not find sunscreen beneficial for their solar urticaria, patients should be recommended to follow sun protection strategies in line with the general population.

Subjective measures of disease activity including patient‐reported outcome measures can usefully evaluate therapeutic response [21], but while the urticaria control test is validated for chronic inducible urticarias [22], there are currently no validated outcome measures specific to solar urticaria. There are also challenges in capturing the impact of an intermittent inducible condition when symptom development is highly dependent on behaviour [5]. However, we found that using the past year DLQI identified substantial impact on patients' QoL pre‐omalizumab therapy, and past week DLQI demonstrated progressive improvement on omalizumab, in line with the increased time spent outdoors and percentage skin exposed, and fewer days with symptoms when outdoors.

Notably, behavioural improvement began during the first month of omalizumab therapy and continued to improve during the rest of spring, summer and autumn. Some authors have reported rapid response to omalizumab in solar urticaria after the first dose [23], whilst others found that response after 2–5 doses could occur [24, 25], and failure to meet primary endpoints in another study at 8 weeks could also reflect the possibility of slower, progressive improvement in some patients [11]. Differences in the onset of response to omalizumab could reflect mechanistic differences: patients with classic “autoallergic” chronic urticaria are expected to respond more rapidly to omalizumab through its direct binding of free IgE and reduction in cell‐bound IgE, while those with “autoimmune IIb” urticaria are expected to respond more slowly, with benefit also obtained through downregulation of FcεR1 receptors on mast cells [21]. Whilst solar urticaria is thought to occur predominantly through “autoallergic” mechanisms and a rapid response would therefore be anticipated, there remain questions about the precise mechanism of action of omalizumab in this context.

Objective measures of disease response include repeat phototesting, with broadband or narrowband provocation testing including determination of the MUD. This can be very helpful in documenting the response, as described in several publications [8, 11, 13, 26], but may not be available in all facilities, particularly narrowband/monochromator testing. Narrowband testing can be particularly useful in providing details of action spectra that influence management strategies, such as personalised approaches to photoprotection based on wavelengths involved. In this series, we demonstrated mostly broad‐spectrum photosensitivity, and all patients showed improvement in MUDs following omalizumab therapy, with many then testing negative at several wavebands. Fold increases in MUDs showed a wide variation, and benefit to individual patients may depend on several factors including baseline MUD, action spectrum and patient lifestyle, thus reinforcing the value of capturing behavioural changes on treatment.

## 6 Limitations

This study was undertaken in a single centre, and given the complexity of data collection and the rarity of solar urticaria, it was conducted in a relatively small number of patients and healthy controls. The patients all had severe solar urticaria, clearly warranting second‐line treatment.

## 7 Conclusions

In conclusion, omalizumab treatment had a positive impact on patients with solar urticaria, enabling them to spend more time outdoors without experiencing debilitating symptoms and to wear more seasonally appropriate clothing. This was associated with significant improvements in quality of life, and further benefits may include improved psychological and emotional well‐being and enhanced vitamin D synthesis. The study reflects not only the efficacy of omalizumab in controlling urticarial symptoms, but also the importance of considering behavioural changes alongside clinical outcomes when assessing the impact of solar urticaria treatment.

## Author Contributions

Conceptualisation: Mark D. Farrar, Lesley E. Rhodes, Kirsty J. Rutter; Data acquisition: Donna Parkin, Elizabeth J. Marjanovic, Mark D. Farrar, Lesley E. Rhodes, Kirsty J. Rutter; Data analysis: Donna Parkin, Elizabeth J. Marjanovic, Kirsty J. Rutter; Data interpretation: Donna Parkin, Elizabeth J. Marjanovic, Mark D. Farrar, Lesley E. Rhodes, Kirsty J. Rutter; Manuscript drafting: Donna Parkin, Elizabeth J. Marjanovic, Kirsty J. Rutter; Manuscript revision: Mark D. Farrar, Lesley E. Rhodes, Kirsty J. Rutter.

## Funding

This work was jointly funded by the National Institute for Health and Care Research Manchester Biomedical Research Centre (Grant NIHR203308), and the British Skin Foundation (Grant 009/BPGSG/18).

## Conflicts of Interest

Lesley E. Rhodes has clinical trials and research collaboration with Mitsubishi Tanabe Pharma America Inc. and Clinuvel Ltd., and Kirsty J. Rutter has research collaboration with Mitsubishi Tanabe Pharma America Inc. and Clinuvel Ltd.; Mark D. Farrar has research collaboration with Clinuvel Ltd. and The Boots Group; these disclosures are all outside the current work.

## Data Availability

The data that support the findings of this study are available from the corresponding author upon reasonable request.
